# Clinical Application of Targeted Next Generation Sequencing for Colorectal Cancers

**DOI:** 10.3390/ijms17122117

**Published:** 2016-12-16

**Authors:** Quitterie Fontanges, Ricardo De Mendonca, Isabelle Salmon, Marie Le Mercier, Nicky D’Haene

**Affiliations:** Department of Pathology, Erasme Hospital, Université Libre de Bruxelles, 1070 Brussels, Belgium; quitterie.fontanges@erasme.ulb.ac.be (Q.F.); ricardo.de.mendonca@erasme.ulb.ac.be (R.D.M.); isabelle.salmon@erasme.ulb.ac.be (I.S.); marie.le.mercier@erasme.ulb.ac.be (M.L.M.)

**Keywords:** targeted next generation sequencing, colorectal cancer, clinical application

## Abstract

Promising targeted therapy and personalized medicine are making molecular profiling of tumours a priority. For colorectal cancer (CRC) patients, international guidelines made *RAS* (*KRAS* and *NRAS*) status a prerequisite for the use of anti-epidermal growth factor receptor agents (anti-EGFR). Daily, new data emerge on the theranostic and prognostic role of molecular biomarkers, which is a strong incentive for a validated, sensitive and broadly available molecular screening test in order to implement and improve multi-modal therapy strategy and clinical trials. Next generation sequencing (NGS) has begun to supplant other technologies for genomic profiling. Targeted NGS is a method that allows parallel sequencing of thousands of short DNA sequences in a single test offering a cost-effective approach for detecting multiple genetic alterations with a minimum amount of DNA. In the present review, we collected data concerning the clinical application of NGS technology in the setting of colorectal cancer.

## 1. Introduction

Colorectal Cancer (CRC) is the second most prevalent cancer in Europe, responsible for 12% of cancer deaths [1]. Despite on-going efforts of developed countries to establish an early preventive screening of the disease, 25% of CRC patients present metastasis at initial diagnosis and 50% will develop metastasis after diagnosis [2]. Patients with unresectable metastatic disease are eligible for targeted therapies such as inhibitors of vascular endothelial growth factor (VEGF) and epidermal growth factor receptor (EGFR) [3]. According to the 2016 European Society for Medical Oncology (ESMO) [3] guidelines, targeted therapies can be used in combination with a cytotoxic regimen for patients with unresectable metastatic CRC.

The classical cytotoxic regimen, used in oncology since the 1960s, works by targeting cells that are in the process of replicating their DNA and does not discriminate cancerous cells from non-cancerous cells, leading to cytotoxicity and major secondary effects [4]. In the late 1980s, the first targeted therapies emerged, which work by targeting cancer-specific molecular pathways that influence the processes that control growth, division, and spread of cancer cells [5]. As described by the National Cancer Institute (NCI) [6], the term therefore encompasses hormone therapies, signal transduction inhibitors, gene expression modulators, apoptosis inducers, angiogenesis inhibitors, immunotherapies, and toxin delivery molecules. Most current targeted therapies are either small molecule drugs or monoclonal antibodies. The emergence of targeted therapies brought huge hopes for both clinicians and patients in clinical oncology, but it was clear from the first trials that this approach was not equally efficient for all patients, underscoring the necessity of identifying predictive biomarkers and ways to implement them into routine practice. In the setting of CRC, targeted therapies are monoclonal antibodies targeting VEGF (Bevacizumab) and EGFR (Cetuximab and Panitumumab) [7]. At present, no clear predictive biomarker has emerged for the use of anti-VEGF agents. In contrast, research studies have discovered more and more potential predictive biomarkers for the use of anti-EGFR agents, as detailed below [8,9,10].

In 2006, Laurent Puig and his group were among the first to demonstrate that *KRAS* exon 2 mutation, which is present in 35%–40% of CRCs, is significantly associated with a poor therapeutic response to the anti-EGFR Cetuximab [8]. In 2010, following this major step and based on the observation that most patients with *KRAS* exon 2 wild-type tumours still did not respond to Cetuximab, further analysis proved that mutations in either *KRAS* (exons 2, 3 and 4) or the closely related *NRAS* (exons 2, 3 and 4) genes were associated with the lack of response to anti-EGFR treatment [9]. About 20% of patients with *KRAS* exon 2 non-mutated tumours harboured one of the extended *RAS* mutations [10]. In a retrospective study performed in 2010, De Roock et al. showed an objective response to Cetuximab of 24.4% in an unselected population, of 36.3% in the *KRAS* wild type population and of 41.2% in the *KRAS*, *BRAF*, *NRAS*, and *PIK3CA* exon 20 wild-type population [9]. More recently, in 2015, Sorich et al. [10] conducted a meta-analysis showing that, for patients treated with anti-EGFR monoclonal antibodies (mAb), the progression-free survival and overall survival were longer for patients without any *RAS* mutations (either *KRAS* exon 2 or new *RAS* mutations) than for patients with *RAS* mutations. Moreover, they found no significant difference in progression-free survival and overall survival between tumours with *KRAS* exon 2 mutations and tumours with the new *RAS* mutations [10]. Biologically, these mutations keep RAS proteins in an active state, leading to constitutive activation of downstream pathways independent of EGFR ligand binding.

In the 2016 ESMO guidelines [3], the expanded *RAS* status appeared as a mandatory prerequisite to the use of anti-EGFR therapy. Moreover, these guidelines also stressed the existence of accumulating evidence that patients with a *BRAF* mutated tumour might not benefit from anti-EGFR therapy, raising the possibility that *BRAF* mutation has predictive value. 

BRAF protein, localized directly downstream of RAS, leads to stimulation of the mitogen-activated protein (MAP) kinase pathway. Accordingly, like for *KRAS*, *BRAF* mutation leads to a constitutive activation of the MAP kinase pathway, ultimately promoting tumourigenesis. *BRAF* mutations (virtually always *BRAF* V600E mutations, which are mutually exclusive with *RAS* mutations) are found in 8% to 12% of metastatic CRCs and yield a dismal prognosis [11]. The testing of *BRAF* mutation status is recommended by the ESMO [3] not only for its prognostic value but also for potential selection for on-going clinical trials. 

In parallel to the RAS/RAF MAP kinase pathway, the PI3K/Akt/mTOR signalling cascade deserves to be mentioned, although not recommended for routine patient management by the latest ESMO guidelines. Indeed, activating mutations in exon 20 of *PIK3CA* (3% of all colorectal cancer) may predict clinical resistance to anti-EGFR monoclonal antibodies, but the correlation is not strong enough to be applied as a routine negative predictive marker. Furthermore, PI3K and its downstream components are attractive targets for inhibition and clinical trials are on-going using PI3K/mTOR inhibitors [12]. Multiple cross talks and negative feedback loops exist between the two pathways and can explain the mechanisms of both acquired and de novo resistance to targeted therapies, therefore they should be seen as a whole. Following this idea, it seems logical that the different mutations that can affect the oncogenic effectors of those pathways are being explored jointly and integrated in gene panels for colorectal cancer next generation sequencing (NGS) testing.

Therefore, the ESMO guidelines highlight the importance of targeted therapy and argue for considering the tumour mutational status in a broader perspective. This approach will deeply impact pathology and genetics laboratories by dictating a new diagnostic paradigm that will result in the shift from low- to high-throughput assays, based on the detection of an increasing number of actionable mutations in a wide variety of tumour types. Diagnostic laboratories are therefore facing several challenges: organizational (workflow, turnaround time), technological, and cost-effectiveness. In this context, the setup of more complex, highly sensitive, high-performing genetic tests, with a limited amount of tissue, within an adequate response time, and at low cost, will be the general rule.

## 2. Next Generation Sequencing

Massively-parallel sequencing or NGS technology appeared at the beginning of the current century as an alternative to Sanger sequencing. Its major advantage is to dramatically increase the sequence throughput by performing several thousands of sequencing reactions simultaneously [13]. This technology has multiple applications, ranging from whole genome sequencing (WGS), through gene expression profiling to a targeted NGS approach, using panels of genes designed to target mutational “hotspots“ of clinical interest. WGS assesses the complete genome of a sample, whereas whole-exome sequencing (WES), which is restricted to the coding regions of the genome (i.e., all exons), generates a limited amount of data compared to WGS. Targeted sequencing uses target-enrichment methods to capture and/or to amplify regions of interest. This approach is becoming increasingly popular in oncology for assessing the mutational status of cancer gene “hotspots”—regions with recurrent mutations. Targeted sequencing enables a deep sequencing, and thus the identification of subclonal mutations. 

NGS technology (among others) was used by international consortia such as The Cancer Genome Atlas (TCGA) Research Network in a multidimensional approach, analysing exome sequences and DNA copy number, identifying epigenetic modifications and depicting the role of microRNA in human cancers, including CRC [14]. These studies provided foundational genetic data and drew the landscape of new theranostic and prognostic molecular biomarkers that needed to be explored and integrated into clinical trials. These data underscored the genetic diversity of colorectal cancer, which was considered as a histopathological homogenous disease. 

The use of WGS for molecular diagnostic pathology is not yet affordable (it is expensive because a high coverage is needed to reach acceptable sensitivity), has a long turnaround time, and requires amounts of DNA that might not be available in clinical practice (especially in the case of small biopsies). In addition, WGS generates a huge amount of data without clear clinical utility (e.g., non-characterized or intronic mutations). However, targeted NGS offers an attractive solution to the new diagnostic paradigm and satisfies the ESMO diagnostic guidelines [3] in terms of efficiency, cost effectiveness and acceptable turnaround time. Gene panels have the advantage of presenting a much higher throughput compared to PCR-based techniques and, depending on the size of the gene panel, they can be cheaper and less time-consuming than PCR or exome and WGS sequencing. Focusing on particular hotspot regions does not provide information on regions outside of the gene panel. On the other hand, fewer data are generated, limiting the need for storage capacity and bioinformatics resources, and considerably facilitating the management of clinically useful genetic information. In terms of sensitivity, gene panels can generate deeper sequencing compared WGS or WES, and could potentially improve mutation detection sensitivity. Additionally, targeted NGS can be performed on a small amount (few nanograms) or even fragmented DNA. This is an important aspect to consider, as in their daily practice, pathologists have to deal with pragmatic limitations, such as: (i) the use of formaldehyde fixation; (ii) small sample sizes, as clinicians favour non-invasive sampling techniques; and (iii) highly variable tumour cell content.

Many publications demonstrated [15,16,17,18] that targeted NGS is suitable for integration into a routine workflow. The applicability of targeted NGS in a routine setting is also illustrated by “basket trials“. In these trials, genomic profiling was used, irrespective of the tumour origin, to propose a targeted therapy. The best example is the NCI MATCH (“Molecular Analysis for Therapy Choice”) study, started in November 2015, which uses a panel of about 143 genes [19]. 

## 3. Clinical Application of NGS to CRC Patients

The aim of our review is to collect the data concerning the clinical application of NGS technology for CRC. The PubMed database was searched for existing English language studies that addressed the use of NGS in colorectal cancer in a clinical setting using the following key words: “next generation sequencing”, “colorectal cancer”, “clinical application”, and ”routine application”. We paid particular attention to the critical factors needed to transfer a testing method into daily clinical practice; i.e., the specimen requirements, the gene panels, performance, turnaround time (TAT) and cost. Concerning specimen requirements, we also tried to focus our scope on studies that used tumour tissue initially sampled in a routine diagnosis perspective and not for research or experimental purposes. By that mean we think that the results obtained were fairly reflecting those that could be expected in a “real” routine clinical setting.

Fourteen studies published between 2013 and 2016 were retrieved (Table 1). Regarding the NGS platform, 10 [18,20,21,22,23,24,25,26,27,28] used the Ion Torrent Personal Genome Machine (PGM) sequencer (Life Technologies), three [29,30,31] used the MiSeq sequencer (Illumina) and one [32] used the Illumina Genome Analyser IIx. 

### 3.1. Specimen Requirements

The development of an assay requiring a small amount of DNA is important for the clinical use, because sometimes the only available samples are specimens with a small amount of material, such as biopsies or fine needle aspirates. For eight out of 10 studies using the PGM sequencer, the required quantity of DNA was 10 ng [20,21,22,23,24,25,27,28]. Two studies assessed that results were obtained using only 0.1 or 0.8 ng of DNA [18,26]. It should be noted that for studies using another platform [30,31,32], the required amount of DNA was higher (up to 3 µg of DNA) than for the PGM platform (Table 1), except for the study of Froyen et al. [29], in which NGS results were obtained with <50 ng of DNA using a custom panel on the Miseq sequencer. In total, 2480 specimens were analysed across the 14 studies; more than 500 of them were biopsies (this information was reported for eight studies [18,21,22,23,24,26,27,29]).

It is of utmost importance that those studies validated their method on FFPE tissue since, despite indisputable efforts of laboratories to save part of the sample for biobanking, fresh frozen material remains uncommon in molecular pathology diagnostics. Among the 14 studies, 13 used FFPE samples and one [32] used fresh frozen primary tumour acquired from a Tumour Bank. However, Han et al. [32] specified that they modified their protocol in order to use FFPE tissue and obtained comparable sensitivity (results not shown). Nevertheless, it should be noted that the 2016 ESMO guidelines [3] recommended that biopsy or tissue sampling procedures should aim to maximize the number of samples collected (ideally *n* = 10 biopsies) and that additional frozen material should be collected to permit future “new” tests to be conducted on frozen tissue if required.

### 3.2. Gene Panels

ESMO recommends the testing of the expanded *RAS* status (*KRAS* exons 2, 3 and 4 and *NRAS* exons 2, 3 and 4) and *BRAF* status [3]. There are the minimum genes for testing CRC. In the analysed studies, the number of analysed genes varied from five to 183. Two studies [23,25] used more than one panel. Commercially available and homemade panels were used in 11 and four studies, respectively (Table 1) (the Ion Ampliseq cancer panel V1 was used once; the Ion Ampliseq cancer panel V2 was used twice; the Ion Ampliseq Colon and Lung panel V1 was used six times; the Ion Ampliseq Colon and Lung panel V2 was used twice; and the Truseq cancer panel was used twice). The number of amplicons per panel varied between 16 and 212 amplicons. 

Altogether, nine different gene panels were used and shared a common core of five genes: *BRAF*, *EGFR*, *KRAS*, *NRAS*, and *PIK3CA*. This core meets the current recommendations of the ESMO for testing the expanded *RAS* status and *BRAF* status. Among this core are also the so-called “emerging biomarkers” that are mentioned in the ESMO recommendations as “not recommended in routine patient management outside of a clinical trials”: *PIK3CA* and *EGFR*. The second most common (eight out of nine panels) group of genes found in the panels included the following four genes: *CTNNB1*, *ERBB2*, *PTEN*, and *SMAD4*. The third most common group of genes (seven out of the nine panel) were the seven following: *AKT1*, *STK11*, *FGFR2*, *ALK*, *MET*, *FBXW7*, and *TP53*. Some of these genes might have been included in the panels because they were not designed exclusively for CRC and the panels might be also used for other tumour types. However, several of these genes are targetable and could also be of interest if found mutated in CRC.

NGS allows a molecular profiling of CRC tumours, permitting the identification of potentially actionable mutations. Jesinghaus et al. [21] reported in their series that 64% of cases have genetic aberrations which potentially influence therapy. However, as already underlined by Meric-Bernstam [33], once the tumour profiling is obtained, one of the hurdles is to perform a clinical trial and to appropriately counsel the patient. Moreover, many variants of unknown significance are found with NGS. Nevertheless, correlations between mutational profiles and clinical response will provide in the future a unique framework for assessing the clinical significance of specific variants [21]. 

### 3.3. Performance

Technologies used for a clinical purpose are expected to have high success rate and to be robust enough to adapt to the inherent variation in size and quality of the samples encountered in routine diagnostic work. For all studies, except the study of Wong et al., less than 10% of the samples failed. The success rate of genetic profiling was excellent overall, varying from 78% to 100% (Table 1). Nevertheless, it should be noted that some of the 100% success rate was reached by selection of samples based on their high DNA content such as in the study of Chevrier et al. [31]. 

The limit of detection (LOD) of the NGS assay was reported in 10 of the 14 studies, and varied from 1% to 8%. For four studies [18,22,24,29], the LOD was validated using serial dilutions of cell lines with known variants (and with known variant frequencies) or using multiplex reference standard carrying different mutations at various defined allelic frequencies. The LOD is a threshold selected as a balance between maximizing the sensitivity and minimizing the false-positive results. This limit of detection had to be compared to the LOD of Sanger sequencing, i.e., 10%–20% [22,26], and LOD of pyrosequencing or real time PCR, i.e., ±5% [26]. Haley et al. estimated that, with a LOD of 10%–20%, Sanger sequencing would have missed 8% of mutations with an allelic frequency <10% or 23% of mutations with an allelic frequency <20%.

The minimum mean coverage to consider a sample acceptable for analysis varies across the studies between 300× and 750× (Table 1).The minimum amplicon coverage to consider an amplicon acceptable for analysis varies across the studies between 100× and 500× (Table 1), except for the study of Han et al. [32] who specified three conditions: (i) the number of uniquely mapped reads at the position should be two or more; (ii) the average base quality (phred Q score) for the position should be 20; and (iii) the read-allele frequency at the position should be 20%. 

### 3.4. Comparison with Other Methods 

Ten of the 14 studies validated the NGS assay by comparing the results for *KRAS* ± *NRAS* ± *BRAF* with an orthogonal method (Sanger sequencing or PCR-based in the majority of the studies). For eight studies, all mutations detected with standard methods were detected with NGS. Moreover, in four studies [18,22,27,31], clinically relevant mutations were found by NGS, but not by the orthogonal method, because the region or the mutation was not covered by the orthogonal method (such as a *KRAS* G13C mutation detected by NGS but not by the Therascreen test in the study of Gao et al. [22]) or the mutation was not detected due to a lower sensitivity of the orthogonal method. Some of these discrepancies were confirmed by a third method (such as digital droplet PCR in the study of D’Haene et al. [18]). Low concordance (67%) was found between NGS and the orthogonal method in the study of Zhang et al. [28]. Recurrent false positive mutations were detected in some genes (*PIK3CA*, *NRAS*, *FGFR2* and *JAK2*). It should be noted that the panel and the data analysis software used in the study of Zhang et al. were in their first versions. Now, new versions are available and were used in the studies of Gao et al. [22] and Haley et al. [26]. In the study of Gao et al. [22], all mutations detected in the *RAS* genes were confirmed by Sanger sequencing. However, Gao et al. specified that detection of a low-frequency mutation by Sanger sequencing is relatively difficult.

### 3.5. Turnaround Time (TAT)

In accordance with recently published ESMO and UK guidelines [3,34], *RAS* testing should be completed and reported within a TAT of ≤7 working days for at least 90% of the test requests. The TAT is defined by ESMO as the time from the receipt of the specimen in the testing laboratory until the report. However, a precise methodological definition of TAT was not given or else varied according to the publication. For instance, TAT can be understood as the time to issuing a final report from: (i) the moment of the clinical request; (ii) the moment of request of histological tissue from its source laboratory; or (iii) the receipt of the tissue at the testing laboratory.

In our review, TAT was addressed by seven of the 14 studies. The reported TAT ranged from 48 h to three weeks, underlining the lack of precise definition of the term. Five out of seven studies [21,23,24,27,29] clarified their definition, TAT being “the period from sample receipt to interpretation of the result” for Malapelle et al. and for Jesinghaus et al. (mean TAT of 13 working days and six days, respectively), or “the time between reception of the sample in the laboratory and report release” for Fontanges et al. (mean TAT of eight calendar days) or “the time from DNA isolation to results” for Tops et al. and Froyen et al. (TAT in between 48 and 72 h). Regardless of the definition used, five of the seven studies that reported their TAT did so within the seven recommended working days [21,23,24,25,29]. Regarding Belardinilli’s study, Froyen’s study and Tops’ study, the reported TAT are, in our understanding, more an estimation of the length of the sequencing workflow (from the start of DNA extraction to the results) but does not take into account the fact that, in clinical daily practice, samples need be pooled and are thus not immediately processed. Indeed, despite the fact that NGS technology allows for a high analysis throughput—multiple samples for multiple genes in a reasonable time—its efficiency is challenged by practical drawbacks: (i) the time for the local pathology lab to prepare and ship the tumour samples to the reference laboratory; (ii) the fact that samples need to be pooled in order to reach a cost-effectiveness threshold; and (iii) the time needed for the results to reach the prescribing clinician. Regarding this aspect, the oncological activity of the hospital is of pivotal importance in combining cost effectiveness and short TAT. 

### 3.6. Cost

Cost is an important factor to consider when implementing a new test with a clinical purpose. Only three studies reported evaluation of the cost of the testing. For the present review, we have also evaluated our own NGS costs [35]. It should be noted that we use the same panels and NGS platform as Belardinilli et al. [25] and Malapelle et al. [27]. Our cost estimations are within the same range as theirs; i.e., 150 to 200 euros of consumable cost per patient. However, it should be noted that this cost is highly dependent on the number of patients tested in each run. The optimal number of samples in one run depends on several parameters such as panel size, average base coverage depth and sequencer throughput. In our own experience, using a 92-amplicon panel on an Ion Torrent PGM with a 318 chip and at an average base coverage of 1000×, 32 samples can be sequenced in one run. In these conditions, and if more than two to three hotspots need to be assessed, NGS testing is cheaper and faster than traditional methods. Belardinilli et al. [25], Tops et al. [24], and Malapelle et al. [27] also report a higher price for traditional methods (Sanger sequencing) than for NGS. For Sanger sequencing, the consumable costs are estimated between 28 and 32 euros per amplicon [24,25,27]. Consequently, the cost of testing of *KRAS*, *NRAS* and *BRAF* (seven exons) is estimated between 196 and 224 euros. Therefore, for a similar cost, targeted NGS sequencing allows a broader tumour profiling.

## 4. Conclusions

New guidelines recommend that, for metastatic CRC patients, the minimum gene regions to test are exon 2, 3, and 4 of *KRAS* and *NRAS* and exon 15 of *BRAF*. Given these recommendations, methods that test the status of multiple genes at once are required. Different molecular assays have been developed for *RAS* and *BRAF* mutation detection in a clinical setting. The requirements for implementation of a new molecular test in daily practice of a pathology diagnostic laboratory include the facts that: (i) the test must be performed on routine samples with low DNA content; (ii) the test results must be delivered rapidly; and (iii) the test results must be accurate and facilitate clinical decision-making. The present review shows that targeted NGS fulfils these requirements and can be successfully applied in clinical daily practice for CRC patients.

Technical validity and clinical utility are the two major issues in the analysis of NGS data. The panel design has to take into consideration the size of the panel (numbers of amplicons) and the clinical utility of the selected genes. Larger panels require longer times for data interpretation and result in higher costs. In the new era of precision medicine, a broader molecular profiling is appealing for the identification of potentially targetable alterations. In this setting, new emerging biomarkers can be added in a NGS gene panel. The main advantage of NGS in comparison to more traditional methods is its capacity to study multiple regions of interests at once. Moreover, the LOD of NGS is higher than for Sanger sequencing (as detailed above). If the cost is calculated per sequenced base, NGS is also cheaper than the other techniques [24]. Implementation of targeted NGS in clinical settings allows for a reliable identification of the most common mutations, which can guide therapeutic decisions for CRC patients. 

## Figures and Tables

**Table 1:** Overview of the studies using next generation sequencing for colorectal cancer patients in a clinical setting.

Article	NGS Platform	Panel	Number of Analysed Genes	Number of Amplicons	Types of Specimen	Number of CRC Samples (Types of Samples)	DNA Quantity	Success Rate	Limit of Detection	Minimum Mean Coverage	Minimum Amplicon Coverage	Concordance with Orthogonal Methods	Cost	TAT
Malapelle et al., 2016 [20]	Ion Torrent PGM	AmpliSeq Colon and Lung Cancer Panel (CLP) V1	22 (hotspots)	90	FFPE	653 NM	10 ng (8 samples with < 10 ng)	100%	5%	NM	100×	NM	NM	NM
Jesinghaus et al., 2016 [21]	Ion Torrent PGM	Custom panel	30 (hotspots)	180	FFPE	202 Surgical resections: 68% Biopsies: 33%	10 ng Min: 6 ng	97%	NM	NM	NM	NM	NM	From sample entry to reporting Mean: 6 days (3–11 days)
Gao et al., 2016 [22]	Ion Torrent PGM	Ion AmpliSeq Cancer Hospot Panel V2	50 (hotspots)	207	FFPE	51 Surgical resections: 36 Biopsies: 15	10 ng	100%	1%	NM	200×	100% *	NM	NM
Fontanges et al., 2016 [23]	Ion Torrent PGM	AmpliSeq Colon and Lung Cancer Panel (V1 and V2)	22 (hotspots)	CLP v1: 90 CLP v2: 92	FFPE	741 Surgical resections: 390 Biopsies: 311 Cytoblock: 7 Not recorded: 33	10 ng	98.1%	4%	500×	250×	NM	NM	From reception of the sample to report release Mean: 8 calendar days
Froyen et al., 2015 [29]	MiSeq	Custom Panel	24 (hotspots)	120	FFPE	40 Surgical resections: ± 50% Biopsies: ± 50%	10–250 ng	90%	5%	300×	300×	100%	NM	From DNA isolation to results: 3 days
D’Haene et al., 2015 [18]	Ion Torrent PGM	AmpliSeq Colon and Lung Cancer Panel	22 (hotspots)	90	FFPE	51 Surgical resections: 44 Biopsies: 7	10 ng (12 cases from 0.1 to 1.5 ng)	100%	4%	500×	250×	100%	NM	NM
Tops et al., 2015 [24]	Ion Torrent PGM	Ampliseq Colon and Lung Cancer Panel V1	22 (hotspots)	87–91	FFPE	59 Biopsies: up to 80%	10 ng	98.3%	4% Hotspots: 2%	500×	500×	100%	130–175 euros/sample	From DNA isolation to results: 48–72 h
Belardinilli et al., 2015 [25]	Ion Torrent PGM	Ion AmpliSeq Colon and Lung Panel (V1 and V2) Custom panel	CLP v1–v2: 22 Custom: 5 (hotspots)	CLP v1: 90 CLP v2: 92 Custom: 16	FFPE	66 NM	10 ng	100%	NM	NM	NM	100%	158–199 euros/sample	4–5 working days
Haley et al., 2015 [26]	Ion Torrent PGM	Ion AmpliSeq Cancer Hotspot Panel V2	50 (hotspots)	207	FFPE	310 Biopsies: 49 Surgical resections: 258 FNA: 3	0.8–30 ng	99.4%	2%	NM	150–500×	NM	NM	NM
Wong et al., 2015 [30]	MiSeq device	TruSeq Cancer Panel (Illumina)	48 (hotspots)	212	FFPE	101	50 ng	78%	8%	750×	100×	97.8%	NM	NM
Malapelle et al., 2015 [27]	Ion Torrent PGM	AmpliSeq Colon and Lung Cancer Panel V1	22 (hotspots)	90	FFPE	114 Surgical resections: 99 Biopsies: 15	10 ng	95.6%	5%	NM	NM	100% *	187.23 euros/sample	From sample entry to results: Mean: 13 working days (7–14 days)
Chevrier et al., 2014 [31]	MiSeq	TruSeq Cancer Panel (Illumina)	48 (hotspots)	212	FFPE	10 NM	>400 ng	100%	NM	NM	NM	100% *	NM	NM
Zhang et al., 2014 [28]	Ion Torrent PGM	Ion AmpliSeq Cancer Panel V1	46 (hotspots)	190	FFPE	22 NM	10 ng	100%	5%	NM	2×	67%	NM	NM
Han et al., 2013 [32]	Illumina Genome Analyser IIx	Custom panel	183 (all exons)	NM	Fresh frozen primary tumour	60 NM	3 µg	100%	NM	NM	NM	100%	NM	Sequencing results were reported within 3 weeks

CLP: Colon and Lung Panel; FFPE: Formalin Fixed Paraffin Embedded; FNA: Fine Needle Aspiration; NM: Not Mentioned; PGM: Personal Genome Machine; TAT: Turnaround Time; * All mutations detected by the orthogonal method were detected by NGS but NGS detected additional mutations.
